# Cell Therapy Using Induced Pluripotent Stem (iPS) Cells Meets Next-Next Generation DNA Sequencing Technology

**DOI:** 10.2174/138920209788921001

**Published:** 2009-08

**Authors:** Manabu Nakayama

**Affiliations:** 1 Department of Human Genome Research, Kazusa DNA Research Institute, 2-6-7 Kazusa-Kamatari, Kisarazu, Chiba 292-0818, Japan; 2 Laboratory of Pharmacogenomics, Graduate School of Pharmaceutical Sciences, Chiba University, 2-6-7 Kazusa-Kamatari, Kisarazu, Chiba 292-0818, Japan

**Keywords:** iPS, next generation DNA sequencing, human genome project, personal genomics, gene therapy, gene replacement therapy.

## Abstract

The recent development of induced pluripotent stem (iPS) cell technology brings cell and gene therapies to patients one large step closer to reality. Technical improvements in various research fields sometimes come together fortuitously, leading to approaches to treating disease. If iPS cell technology continues to progress smoothly as expected and is actually applied to patients, the next logical step to ensuring the success of iPS cell therapy is to make use of next-next generation DNA sequencing technology and bioinformatics of recipient genomes. Before a patient-derived iPS cell colony is used for clinical therapy in a patient, the colony should undergo whole-genome DNA sequencing, thus avoiding risks associated with spontaneously mutagenized iPS cells. Researchers participating in the Human Genome Project need to take full advantage of both technologies—iPS cell technology and DNA sequencing—as doing so will help us achieve the original long-term goal of the project: developing therapies that will benefit human health.

Induced pluripotent stem (iPS) cells can be produced by introducing four transcriptional factors—Oct4, Sox2, Klf4, and c-Myc—into embryonic or adult somatic cells by means of a retroviral vector [1]. The retroviral vector system allows the efficient and simultaneous transfection of the four transcription factors into primary cultured cells, enabling them to continuously express foreign genes for more than two weeks. Because iPS technology possesses great potential for a wide range of applications, including the development of therapies for diseases, several researchers worldwide have tested its reproducibility and have improved the technology extensively [2]. During this refinement, researchers had to overcome several challenges, such as how to reprogram human somatic cells to become pluripotent cells [3, 4]; and how to produce iPS cells through non-integrating gene-delivery systems [5, 6]. Initially, retrovirus vectors were integrated into mouse and human genomes randomly, which is necessary for the continuous expression of the four reprogramming factors. Random integration of the vector, however, may activate or disrupt genes located near the integration site, leading to tumorigenicity. To solve this problem, researchers have recently developed an adenoviral vector [5] and repeated transfection protocol of expression plasmids [6]. Despite these efforts, the possibility that a piece of the vector will integrate into the recipient cells cannot be excluded.

Currently, the hottest competition in research and the drug-discovery field involves the identification of small molecules that can replace potentially harmful factors involved in the induction process [7-11], such as c-Myc, a well-known oncogene, and Klf4, a potential oncogene. The identification of additional small molecules is now on the horizon, as the individual roles Oct4, Sox2, Klf4, and c-Myc play in converting somatic cells into pluripotent cells have recently been clarified [12]. Nonetheless, it remains unknown whether such small molecules will be safer in the long-term than the four reprogramming factors.

Another problem daunting current iPS cell methodologies is that the long-term incubation of primary cultured cells needed to generate iPS cells may also cause unexpected mutations to accumulate in the iPS cell genome. In fact, recent analyses of human embryonic stem (hES) cells cultured over long periods have reported recurrent genomic instability in the human genome [13, 14]. Thus, before patient-derived iPS cells are clinically applied to the patient, the iPS cells need to be screened for unexpected mutations. This can be achieved by subjecting the cells to whole-genome DNA sequencing.

Since the completion of the Human Genome Project, genome researchers have shifted their interest to personal genomics. Next and next-next generation DNA sequencing permits the re-sequencing of entire human genomes in a short time and at low costs [15, 16]. Pacific Biosciences will soon make commercially available a large-scale DNA sequencer capable of performing real-time DNA sequencing from single polymerase molecules [17]. Many researchers in both academia and biotechnology are pursuing the “$1,000 genome” using single-molecule approaches [18]. Another company, Complete Genomics, recently announced its plans to start offering a human genome sequencing service for companies and academic institutions in 2009, charging $5,000 per genome. State-of-the-art breakthroughs such as next-next generation DNA sequencing will enable us to rapidly assess the entire genome of autologous, patient-derived iPS cells before they are used in patients. This will reduce the risk of introducing artificial mutations into the patient.

The low efficiency of current iPS cell methods can be avoided by using human keratinocytes derived from patients’ hair follicles and reprogramming them into iPS cells, a process which has shown to be rapid and highly efficient [19]. Using keratinocytes is very advantageous in that patients experience fewer burdens, as keratinocytes are isolated by plucking a patient’s hair. In the future, even patients with genetic disorders will receive cell and gene therapies, although they might benefit from iPS technology first. Indeed, treating these patients with gene therapy is imminent, as the principle underlying the treatment of genetic disorders has already been demonstrated through the use of homologous recombination and gene-targeting techniques to repair a gene with a disease-specific mutation in human iPS cells [20]. As iPS technology continues to progress rapidly, we can expect that, in the near future, iPS cells derived from keratinocytes will be generated by using only small molecules, replacing the four transcription factors, or by using a non-integrating gene-delivery method of introducing a minimal number of reprogramming factors.

Generally, several iPS colonies are produced during the initial stages of reprogramming. Clinicians, however, usually select only one iPS colony before differentiating the cells to the needed cell type (e.g., hemocyte, pancreatic β-cell, cardiac muscle cell, or neuron) and introducing the cells into patients. By determining the entire DNA sequence of some iPS colonies and comparing the sequence with the DNA sequence of the patient’s genome, clinicians can confirm iPS cell quality and select cells that lack mutations and/or those not susceptible to unexpected genomic changes. Cells containing spontaneous mutations, unexpected genomic changes, or pieces of vector integrated into the genome can be identified and discarded, thus averting potential risks of tumorigenicity and unpredictable results. Before the advent of rapid, large-scale DNA sequencing technologies, the criteria used by researchers and clinicians to select an iPS cell colony for future transplantation was whether the cells appeared healthy. Unlike this intuitional selection method, whole-genome DNA sequencing of iPS cells for clinical use provides a solid way to select iPS cells intended for transplantation, safeguarding recipient patients from the potential risk of tumor formation.
